# Identification of a Novel Antigenic Epitope in Envelope Protein of Avian Reticuloendotheliosis Virus

**DOI:** 10.3390/vetsci13030263

**Published:** 2026-03-12

**Authors:** Jingzhe Han, Mengmeng Huang, Guodong Wang, Yulong Zhang, Runhang Liu, Hangbo Yu, Ziwen Wu, Erjing Ke, Dan Ling, Suyan Wang, Yuntong Chen, Yongzhen Liu, Yanping Zhang, Hongyu Cui, Yulu Duan, Liuan Li, Yulong Gao, Xiaole Qi

**Affiliations:** 1 State Key Laboratory of Animal Disease Control and Prevention, Avian Immunosuppressive Diseases Division, Harbin Veterinary Research Institute, The Chinese Academy of Agricultural Sciences, Harbin 150069, China; 13132030859@163.com (J.H.); huangmm1017@163.com (M.H.); setback1231@163.com (G.W.); yulo_zh@163.com (Y.Z.); lrh18730280216@163.com (R.L.); yuhangbo2022@163.com (H.Y.); 17590395515@163.com (Z.W.); elisake0924@163.com (E.K.); qingxiqingxi0831@163.com (D.L.); wangsuyan@caas.cn (S.W.); chenyuntong@caas.cn (Y.C.); liuyongzhen@caas.cn (Y.L.); zhangyanping@caas.cn (Y.Z.); cuihongyu@caas.cn (H.C.); duanyulu@caas.cn (Y.D.); 2 Tianjin Key Laboratory of Agricultural Animal Breeding and Healthy Husbandry, College of Animal Science and Veterinary Medicine, Tianjin Agricultural University, Tianjin 300392, China; 3 Heilongjiang Province Key Laboratory of Veterinary Immunology, Harbin Veterinary Research Institute, The Chinese Academy of Agricultural Sciences, Harbin 150069, China; 4 Jiangsu Co-Innovation Center for the Prevention and Control of Important Animal Infectious Disease and Zoonosis, Yangzhou University, Yangzhou 225009, China

**Keywords:** avian reticuloendotheliosis virus, gp90, protein expression, monoclonal antibody, antigenic epitope

## Abstract

Avian reticuloendotheliosis (RE) caused by reticuloendotheliosis virus (REV) is an important tumor and immunosuppressive disease posing a serious threat to poultry development. The REV envelope protein, glycoprotein (gp)-90, not only participates in cell receptor binding and viral assembly and release but also induces neutralizing antibody production. However, the antigenic epitope structure of gp90 has not yet been systematically understood. Therefore, in this study, the gp90 envelope protein of a predominant REV strain was prepared using a prokaryotic expression system, and a hybridoma cell line stably secreting the REV gp90 monoclonal antibody was developed via cell fusion and flow cytometry. Notably, a novel linear B-cell epitope, ^195^REESVRERL^203^, was identified for the first time in the gp90 of REV using peptide scanning. This epitope was located on the outer side of the gp90 midpiece and was conserved across REV strains. Overall, this study is of great significance for the systematic understanding of REV antigen structure and the development of virus detection methods.

## Simple Summary

Reticuloendotheliosis is a devastating poultry disease causing tumors and immunosuppression in infected birds. Reticuloendotheliosis virus (REV) encodes a surface glycoprotein (gp90) that mediates viral infection, assembly, release and induces protective antibodies. However, the specific gp90 regions triggering immune responses have not been fully characterized. In this study, we expressed gp90 of a prevalent REV strain using a prokaryotic expression system and established a hybridoma cell line stably secreting gp90-specific monoclonal antibodies through cell fusion and flow cytometric screening. Most notably, we identified a novel conserved linear B-cell epitope, ^195^REESVRERL^203^, on the outer side of gp90 for the first time. This study is of great significance for the systematic understanding of REV antigen structure and the development of virus detection methods.

## 1. Introduction

Avian reticuloendotheliosis (RE) is caused by the avian reticuloendotheliosis virus (REV), which is also associated with other diseases and conditions, including acute reticuloendotheliosis, chronic lymphoid tumors, and short stature syndrome, resulting in large-scale death or growth retardation in chickens, posing a major threat to the poultry industry [1,2]. REV, which is transmitted both horizontally and vertically, easily co-infects birds with the avian leukosis virus and Marek’s disease virus, resulting in severe immunosuppression [3,4]. This increases birds’ susceptibility to other pathogenic microorganisms and causes significant losses to the poultry industry [5,6]. It is worth noting that wild birds can also be infected with REV [7,8]. Therefore, new broad-spectrum diagnostic tools based on highly conserved epitopes are urgently needed to combat the rising REV prevalence, prevent vaccine contamination, and address the critical gaps in rapid diagnostic technologies for field applications [9,10,11].

REV, belonging to the *Gammaretrovirus* genus in the *Retroviridae* family, possesses a positive-sense single-stranded RNA genome. The two single strands RNA usually form a dimeric structure, which is of great significance for the correct genome replication and viral assembly of REV. REV genome contains the group-specific antigen (gag), polymerase (pol), and envelope (env) genes and two long terminal repeats [12,13]. The precursor protein encoded by env is cleaved into glycoprotein (gp)-20 (transmembrane protein) and gp90 (surface envelope protein) [14,15]. The gp90 protein is a key protein mediating REV invasion into host cells. It participates in cell receptor binding and viral assembly and release and induces neutralizing antibody production [16,17,18,19]. Moreover, gp90 is a key target for the development of new diagnostic reagents and vaccines. Monoclonal antibody (MAb)-based peptide scanning is a traditional method to identify novel antigenic epitopes. Since the pioneering development of 11 MAbs targeting both strain-specific and common REV epitopes by Cui et al. [20], research on REV gp90 MAbs has progressively increased [21,22,23]. However, the complete antigenic epitope structure of gp90 is unknown.

In this study, a MAb against REV gp90 was developed and a novel antigenic epitope recognized by this MAb was identified, providing a basis for further analysis of the REV gp90 antigenic structure.

## 2. Materials and Methods

### 2.1. Cells, Viruses, Plasmids, and Strains

Mouse myeloma cells (SP2/0), DF-1 cells, chicken embryo fibroblasts (CEFs), and eukaryotic expression vector pCAGGS were preserved by the Avian Immunosuppressive Disease Division (hereafter referred to as our laboratory), Harbin Veterinary Research Institute (HVRI), and Chinese Academy of Agricultural Sciences (CAAS). DF-1 cells were cultured in the Dulbecco’s minimum essential medium (DMEM) containing 10% fetal bovine serum (43640C; Sigma, Burlington, MA, USA) at 38.5 °C and 5% CO_2_. CEFs and SP2/0 cells were cultured in DMEM containing 10 and 20% fetal bovine serum, respectively, at 37 °C and 5% CO_2_. Representative REV strain HLJR0901 was previously isolated and identified in our laboratory [24]. The recombinant plasmid pBlu-HLJR0901, containing the full-length genome of HLJR0901, and recombinant eukaryotic expression plasmid pCAGGS-gp90, containing the envelope protein gene gp90 of HLJR0901, were constructed in our laboratory. Prokaryotic expression vector pCold-I (+) and engineered bacterium BL21 (DE3) were purchased from TaKaRa Biotechnology (Dalian, China) Co., Ltd.

### 2.2. Expression of REV gp90 Protein

REV gp90 gene was amplified from the pBlu-HLJR0901 recombinant plasmid using primers containing *Eco*RI and *Xho*I sites (forward primer pCold1-gp90-F: 5′-TCGAAGGTAGGCATATGGAGCTCGGTACCCTCGAGCATCATCATCATCATCATGACTGTCTCACCAACCTCCGATCCGCTGAGGGTAA-3′; reverse primer pCold1-gp90-R: 5′-ACCTATCTAGACTGCAGGTCGACAAGCTTGAATTCTTACTTATGACGCCCAGCGGTGTACTCGATGGATG-3′), and the amplified fragment (1.3 kb) contained the full-length gp90 gene with an N-terminal His-tag. The amplified gp90 gene was further cloned into the prokaryotic expression vector pCold-I using a homologous recombination kit (C115-1; Vazyme, Nanjing, China), and the recombinant plasmid with the correct sequence was transformed into BL21 (DE3) for gp90 expression. The bacterial culture in the logarithmic growth phase was used, and 1 mmol/L isopropyl β-D-1-thiogalactopyranoside (IPTG) was added to induce protein expression at 20 °C for 22 h. The bacterial cells were disrupted using an ultrasonic cell disruptor (Sonic), and whole cells, supernatants, and precipitates were collected to prepare the protein samples. His-tagged recombinant proteins were detected via sodium dodecyl sulfate-polyacrylamide gel electrophoresis (SDS-PAGE) and purified via gel elution: the samples were centrifuged at 12,000× *g* for 30 min at 4 °C, the supernatant was discarded and the pellet was re-suspended in PBS, followed by adding 5 × Loading Buffer and boiling in a water bath for 10 min for SDS-PAGE; the gel was then stained with 0.25 mol/L KCl solution, the target bands were excised when appearing silvery white and decolorized in ultrapure water on a shaker until the gel strips were completely transparent. Then the proteins were purified by gel elution at 250 V for 25 min. Protein concentration was determined using a microplate reader (Implen) and stored at −80 °C for future use.

### 2.3. Preparation of REV gp90 MAb

Purified recombinant protein gp90 was mixed and emulsified with the Freund’s complete adjuvant (F5881; Sigma, Burlington, MA, USA) in equal volumes and subcutaneously injected into five six-week-old female BALB/c mice (0.1 mg/mouse). Subsequently, the mice were immunized four times with the purified gp90 emulsified in Freund’s incomplete adjuvant, with a two-week interval. Two weeks after each immunization, tail tip blood was collected and serum antibody titers were detected using an indirect enzyme-linked immunosorbent assay (ELISA) coated with immunogens. Seven days after the fifth immunization, the mice with the highest antibody titers were selected and intraperitoneally injected with 0.1 mg of gp90 protein for booster immunization. Three days later, in the cell fusion experiment, spleen cells were isolated from euthanized mice and fused with SP2/0 cells mediated by polyethylene glycol (P7306; Sigma). The fused cells were subsequently cultured in HAT selective medium (Sigma) containing hypoxanthine aminopterin thymidine (H0262; Sigma) to screen hybridoma cells. Ten days later, antibody titers of the hybridoma cells supernatant were determined via indirect ELISA. Positive cells were subjected to two rounds of subcloning using a flow cytometry cell sorting system (Sony, San Jose, CA, USA). For subcloning, aliquots of hybridoma cells were added to flow cytometry tubes, and single cells were sorted into 96-well plates containing HT medium (200 μL/well; Sigma) at one cell per well. The gp90 MAb-secreting cells were screened using indirect ELISA and the selected monoclonal hybridoma cell lines were cultured in DMEM containing 20% fetal bovine serum. Furthermore, the MAb ascites were prepared. Ten healthy six-week-old female BALB/c mice were intraperitoneally injected with the Freund’s incomplete adjuvant. After 5–7 d, monoclonal hybridoma cells at the logarithmic growth stage were injected into the abdominal cavities of mice. Tumor occurrence was monitored daily and MAb ascites were collected from the swollen abdomen of mice at different time points after 7 days. The collected ascites were centrifuged at 10,000× *g* for 20 min at 4 °C. The ascites were carefully extracted from the middle layer, aliquoted, and stored at −80 °C for future use. All animal experiments (approval number: 240711-02-GR) were approved by the Institutional Animal Care and Use Committee (IACUC) of HVRI.

### 2.4. Identification of Antigenic Epitope of REV gp90

To identify the antigenic epitope recognized by the REV gp90 MAb C16, gp90 gene of REV HLJR0901 strain was truncated and cloned into the pCAGGS plasmids to construct recombinant eukaryotic expression plasmids. Subsequently, the recombinant eukaryotic expression plasmids were transfected into DF-1 cells to express truncated gp90 using TransIT-X2 Dynamic Delivery system (Mirus Bio LLC, Madison, WI, USA) according to its product manual. Thirty hours after transfection, cell samples were collected for Western blotting analysis. The gp90 epitopes were screened and identified based on the reaction between gp90 MAb C16 and gp90 truncate. The construction scheme of gp90 truncation is as follows. First, we split gp90 into three overlapping segments: P1 (aa 1–137), P2 (aa 133–269), and P3 (aa 265–397). We further divided P2 into four fragments: P2-1 (aa 133–169), P2-2 (aa 165–204), P2-3 (aa 200–236), and P2-4 (aa 232–269). Next, we truncated P2-2 into six fragments: L1 (aa 165–194), L2 (aa 165–184), L3 (aa 165–174), R1 (aa 175–204), R2 (aa 185–204), and R3 (aa 195–204). Finally, we shortened R3 into six fragments: L9 (aa 195–203), L8 (aa 195–202), L7 (aa 195–201), R9 (aa 196–204), R8 (aa 197–204), and R7 (aa 198–204). All truncated bodies carried the enhanced green fluorescent protein tag gene at their N-terminus.

### 2.5. Western Blotting

DF-1 cells were lysed with the 1% NP-40 lysis buffer (ST366; Beyotime, Shanghai, China), and the cell lysates were separated via SDS-PAGE. Subsequently, the proteins were transferred onto nitrocellulose membranes and blocked with 5% skim milk at room temperature for 1 h. After washing three times with PBST (Phosphate-Buffered Saline with 0.1% Tween-20, pH 7.4), the membranes were incubated with specific primary antibodies, including the REV gp90 MAb C16, mouse anti-His (1305538; Sigma), anti-enhanced green fluorescent protein (MA1-952; Invitrogen, Carlsbad, CA, USA), anti-Flag (F1804; Sigma), and anti-beta-actin (A1978; Sigma) antibodies, at room temperature for 1 h. After washing four times with PBST, the membranes were incubated with the IRDye 800CW goat anti-mouse IgG antibody at room temperature for 45 min. Finally, after washing four times with PBST, the membranes were scanned using the Odyssey Infrared Imaging System (LiCor Biosciences, Lincoln, NE, USA).

### 2.6. ELISA

Recombinant gp90 protein was diluted to 10 μg/mL with a carbonate coating solution, added to a 96-well ELISA plate at 100 μL/well, and incubated overnight at 4 °C. After washing five times with PBST, the wells were blocked with 5% skim milk (100 μL/well) and incubated at 37 °C for 1 h. After washing, the diluted serum samples (1:1000 to 1:64,000; 100 μL/well) were added to the wells and incubated at 37 °C for 1 h. The wells were then washed five times with PBST. After adding the rabbit anti-mouse IgG coupled with horseradish peroxidase (1:3000; A4416-mL; 100 μL/well; Sigma), the samples were incubated at 37 °C for 1 h, washed five times, and incubated with a soluble single-component tetramethylbenzidine solution (PA107; TIANGEN, Beijing, China) for color development (100 μL/well) at 37 °C for 15 min. The reaction was terminated by adding 2 M H_2_SO_4_ (100 μL/well), and the plate was immediately placed on a fully automated ELISA microplate reader (BioTek, Winooski, VT, USA) to determine the optical density at 450 nm.

### 2.7. Indirect Immunofluorescence Assay (IFA)

Recombinant expression plasmid was transfected into DF-1 cells for 30 h, or REV was inoculated into CEFs for seven days. After washing twice with PBS, the cells were fixed with 4% paraformaldehyde at room temperature for 20 min. After washing thrice, the cells were blocked with 5% skim milk at 37 °C for 1 h, washed five times, and incubated with the REV gp90 MAb C16 primary antibody at 37 °C for 1 h. After washing five times, the cells were incubated with fluorescein 5-isothiocyanate-labeled goat anti-mouse IgG antibody (926-32210; Sigma) at 37 °C for 45 min. Finally, after five washes, the cells were examined under an inverted fluorescence microscope (AMG, Seattle, WA, USA).

### 2.8. REV gp90 MAb Subtype Identification

IgG subclass of gp90 MAb was analyzed using the Mouse Monoclonal Antibody Isotyping ELISA Kit (Biodragon, Beijing, China), according to the manufacturer’s instructions.

### 2.9. REV gp90 Antigenic Epitope

AlphaFold3 (https://alphafoldserver.com/accessed on 20 December 202) was used to predict and analyze the three-dimensional structure of the REV HLJR0901 gp90 protein. PyMOL (Version 3.2; Schrödinger, New York, NY, USA) was used to analyze the spatial position and amino acid characteristics of the antigenic epitope recognized by MAb C16. Representative REV strains were selected to analyze the conservation of this antigenic epitope. All strains and their GenBank accession numbers are listed in Table 1.

## 3. Results

### 3.1. Recombinant REV gp90 Protein Expression and Purification

**Figure 1:**
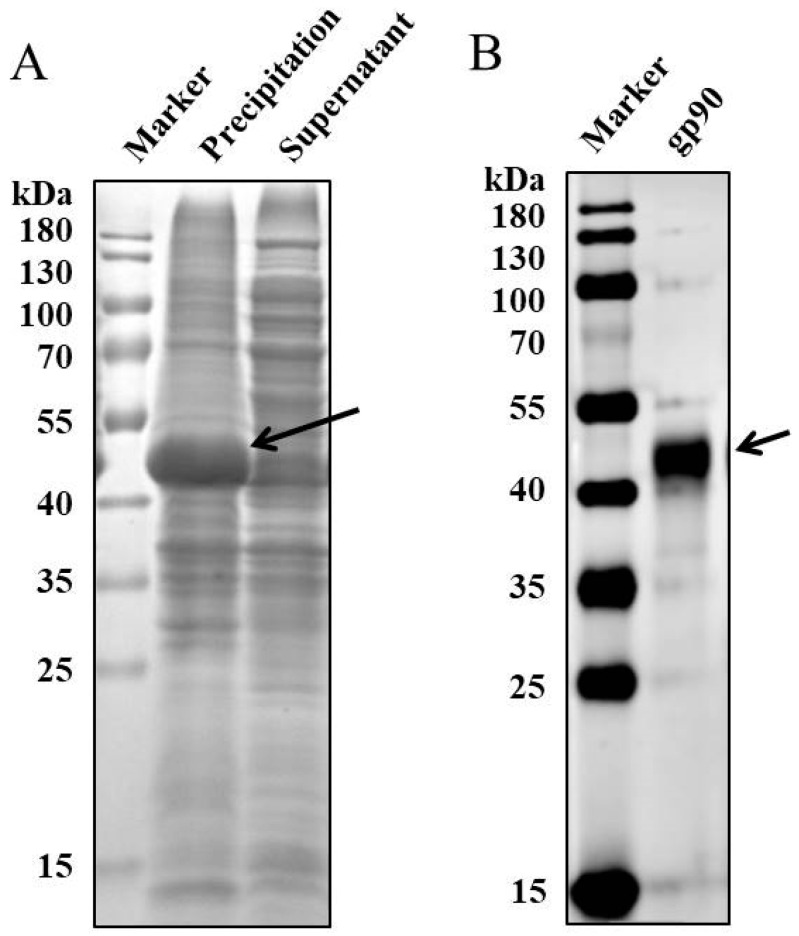
Expression and identification of recombinant gp90 of REV. (**A**) Recombinant gp90 expression as an inclusion body in BL21 (DE3) cells analyzed via SDS-PAGE. (**B**) Western blotting identification of purified recombinant protein gp90 using an anti-His antibody. The recombinant protein gp90 was highlighted with an arrow.

The supernatant and bacterial precipitation samples were prepared after ultrasonic treatment, and recombinant protein expression in bacteria was detected via SDS-PAGE. The recombinant protein gp90, with a molecular weight of approximately 46 kDa, was mainly expressed as inclusion bodies (Figure 1A) and purified via gel electroelution. Western blotting revealed that the purified recombinant protein gp90 exhibited good specificity for the mouse anti-His antibody (Figure 1B).

### 3.2. REV gp90 MAb Preparation and Identification

**Figure 2:**
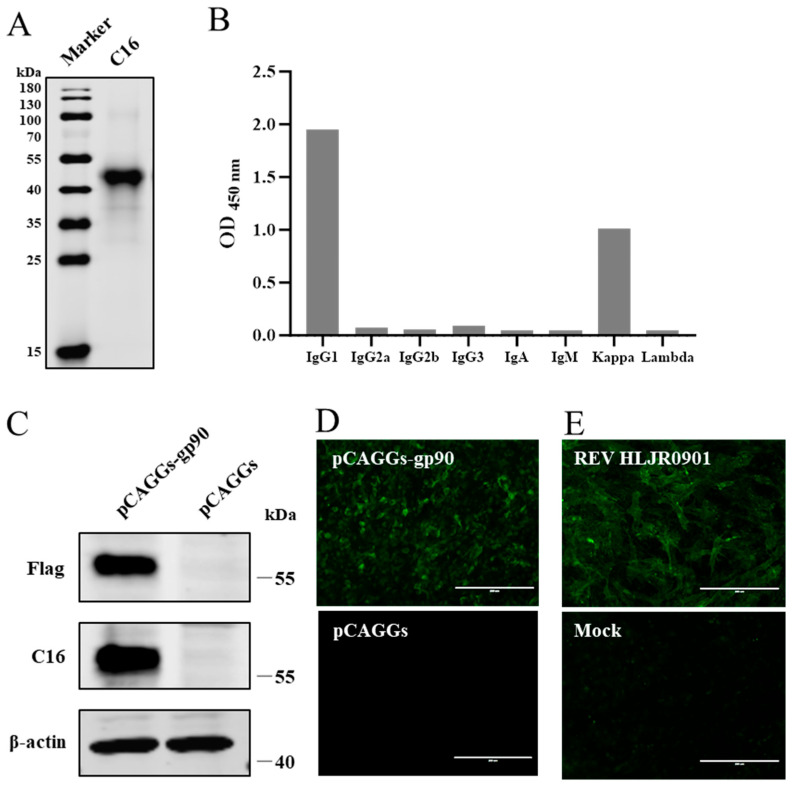
Identification of the REV gp90 MAb C16. (**A**) Reactivity of MAb C16 with the recombinant prokaryotic expression protein gp90 assessed via Western blotting. (**B**) MAb subtype analysis using the Mouse Monoclonal Antibody Isotyping ELISA Kit. (**C**,**D**) Reactivity of MAb C16 with the recombinant gp90 protein expressed by the eukaryotic expression plasmid assessed via Western blotting (**C**) and IFA (**D**). (**E**) Reactivity of MAb C16 with REV in CEFs assessed via IFA.

A hybridoma cell line secreting REV gp90 MAb was identified via cell fusion and flow cytometry and named C16. Western blotting revealed that MAb C16 exhibited good reactivity with both the recombinant prokaryotic (Figure 2A) and eukaryotic (Figure 2C) gp90 expression proteins. The anti-Flag and anti-β-actin antibody was used as control, respectively. The prokaryotically expressed His-tagged gp90 protein exhibited a molecular weight of approximately 46 kDa, whereas the eukaryotically expressed Flag-tagged gp90 protein exhibited a molecular weight of approximately 60 kDa. In IFA with MAb C16, both the pCAGGS-gp90 transfection and REV infection groups showed specific green fluorescence, whereas the pCAGGS (Figure 2D) and mock (Figure 2E) groups showed no fluorescence. Notably, MAb C16 was identified as an IgG1 antibody with a kappa chain (Figure 2B).

### 3.3. Identification of the Antigenic Epitope Recognized by MAb C16

**Figure 3:**
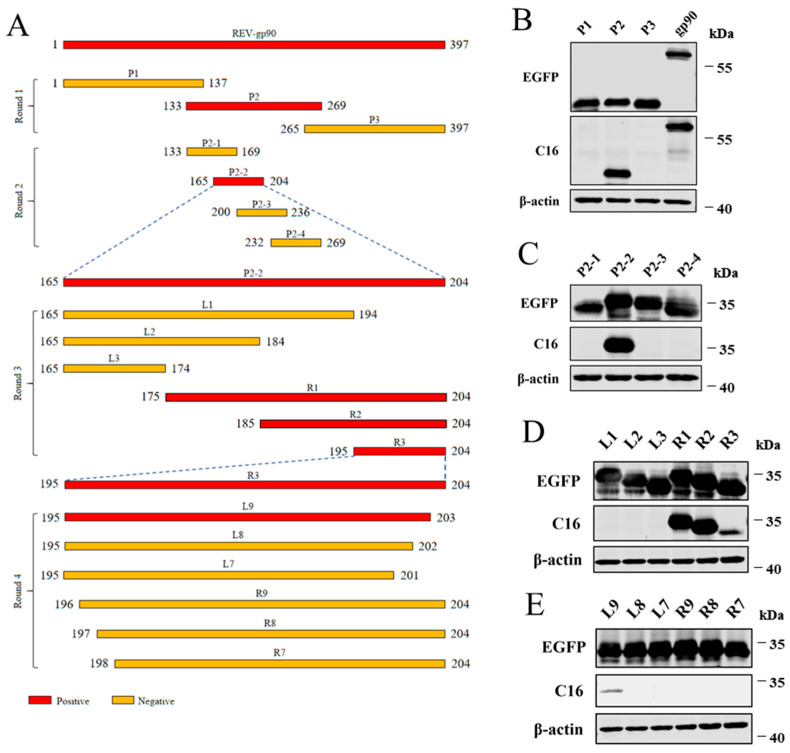
Antigenic epitope mapping using gp90 MAb C16. (**A**) Schematic diagram of REV gp90 epitope mapping. Fragments of gp90 recognized by MAb C16 are highlighted in red. (**B**–**E**) Identification of the gp90 epitope recognized by MAb C16 via Western blotting.

Western blotting analysis of all truncated bodies of gp90 (Figure 3) showed that P2 (aa 133–269) was specifically recognized by MAb C16 in the first round of detection (Figure 3B). In the second round of detection, MAb C16 further recognized P2-2 (aa 165–204; Figure 3C). In the third round of detection, MAb C16 reacted with R3 (aa 195–204; Figure 3D). Final truncated expression of R3 was performed to precisely locate the shortest epitope recognized by C16. As shown in Figure 3E, MAb C16 specifically recognized L9, indicating that ^195^REESVRERL^203^ is one antigenic epitope of gp90 specifically recognized by MAb C16.

### 3.4. Visual Analysis of the Antigenic Epitope

**Figure 4:**
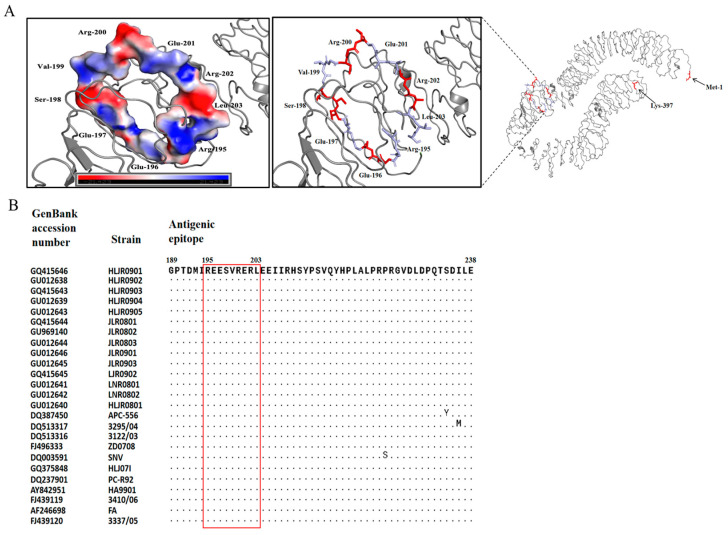
Sequence and amino acid characteristics analysis of the epitope recognized by gp90 MAb C16. (**A**) Spatial distribution and surface electrostatic potential map (negative or positive electrostatic potential is represented by red or blue) of the identified epitope on the predicted three-dimensional (3D) gp90 structure. (**B**) Amino acid sequence alignment of the identified epitope (red box) in different REV strains.

Structural visualization revealed that the ^195^REESVRERL^203^ epitope was located on the outer side of the gp90 protein midpiece and formed a ring-like structure (Figure 4A). This epitope contains three positively charged arginine residues (Arg-195, Arg-200, Arg-202) and three negatively charged glutamic acid residues (Glu-196, Glu-197, Glu-201), which are arranged in a staggered manner on the ring. This complementary charge distribution may form a local electrostatic network, which is beneficial for the electrostatic attraction between MAb and epitope. In addition, neutral residues such as Ser-198, Val-199, and Leu-203 exist at the transition positions in the charge region. The hydrophobic side chains of Val-199 and Leu-203 may have hydrophobic interactions with MAb, while the polar side chains of Ser-198 can participate in the formation of hydrogen bonds (Figure 4A). Sequence alignment indicated that the gp90 antigenic epitope ^195^REESVRERL^203^ was conserved across REV strains (Figure 4B).

## 4. Discussion

REV is among the three major tumor pathogens posing serious threats to the global poultry industry by causing immunosuppression and a decline in the overall production performance [25,26,27]. As REV is a retrovirus, use of its vaccine is not advocated in many countries. Virus purification and elimination remain the primary RE prevention and control measures. However, because of the huge investment cost, implementation of these measures faces significant challenges, especially in developing countries, making the long-term threat of REV difficult to resolve. Therefore, developing safe and reliable REV detection methods and strict monitoring of REV pollution in biological products are necessary for RE prevention and control. As the surface envelope protein of REV, gp90 is indispensable for RE clinical detection, and its MAb preparation and antigenic epitope identification are important for accurate REV detection.

MAbs are necessary tools for basic and applied viral research. To develop the REV gp90 MAb, gp90 genes were extracted from the representative REV strains. During protein production, upon testing different parameters, we determined that the optimal conditions for achieving maximum inclusion body expression were 20 °C for 22 h with 1 mmol/L IPTG. Positive gp90 MAb-secreting hybridoma cells were obtained by fusing spleen cells from immunized BALB/c mice with SP2/0 myeloma cells. For further hybridoma cell purification, two rounds of flow cytometry sorting were performed to identify a single hybridoma cell line stably secreting the gp90 MAb. Traditionally, the screening of positive hybridoma cells is performed using the limited dilution method. However, the errors in manual cell counting and the complexity of limited dilution operations can sometimes lead to lower experimental efficiency. The flow cytometry can accurately sort individual cells, greatly improving the sorting speed and positivity rate of hybridoma cells [28]. When preparing MAb ascites, both Freund’s incomplete adjuvant (FIA) and pristane can be used to sensitize BALB/c mice (Jones et al., 1990; Kints et al., 1989) [29,30]. Our laboratory often uses IFA [28], which has good effects. The gp90 MAb C16 obtained in this study exhibited good specificity for the gp90 protein and accurately detected it in IFA and Western blotting.

To further analyze the antigenic structure of gp90, peptide scanning was performed to identify the antigenic epitopes recognized by the REV gp90 MAb C16. ^195^REESVRERL^203^ was identified as the key unit recognized by gp90 MAb C16. Our data in Figure 3 show that MAb C16 recognizes R3 (aa 195–204) and L9 (aa 195–203), but not L8 (aa 195–202), suggesting that ^195^REESVRERL^203^ is the shortest core epitope recognized by MAb C16. Additionally, we noticed the stronger protein binding of R2 (aa 185–204) than R3 (aa 195–204) to MAb C16, suggesting that residues near the core epitope promote MAb C16 binding to the core epitope; however, the specific molecular mechanisms require further research. Residue 200–245 of gp90 is a large antigenic epitope region, in which ^213^SVQYHPL^219^, ^216^YHPLA^220^, and ^230^DPQTSDILEA^239^ are recognized by the corresponding MAbs [21,22,31]. Notably, this study identified a new antigenic epitope (^195^REESVRERL^203^) at the N-terminal overlapping position of this large antigenic epitope region. The predicted ring-like structure of ^195^REESVRERL^203^ is possibly beneficial for antibody binding, warranting further investigation. Moreover, the specific relationship between these epitopes remains unknown. The specificity of an antigenic epitope is determined by its amino acid composition, with some residues being more important for antibody binding than others [21]. As it is the main protective antigen of REV, further research is necessary to gain a more systematic understanding of the epitope structure of gp90 antigen.

The neutralization activity of MAb C16 has also been detected, which showed that MAb C16 cannot effectively neutralize REV. Nevertheless, the identification of the novel linear B-cell epitope provides critical theoretical and technical support for further exploration of REV antigen structure and establishment of novel detection methods. Notably, our identified epitope ^195^REESVRERL^203^ is conserved across REV strains, potentially serving as a core target for diagnostic tests to improve the specificity and broad spectrum of REV detection. For example, single or multiple epitope peptides can be used as coated antigens for the development of ELISA for REV antibody detection [32]. In addition, MAb with broad-spectrum reactivity can be used for the development of REV or antigen tracing detection methods such as IFA. Although sequence alignment confirmed the conservation of the gp90 antigenic epitope ^195^REESVRERL^203^, it is necessary to conduct more detailed research on the specificity and intensity of antigen–antibody reaction before formally developing relevant detection methods using the MAb C16 and its recognized epitope. These experiments will be carried out in our subsequent studies.

In this study, we prepared the envelope protein gp90 of a predominant REV strain using a prokaryotic expression system. Additionally, we generated a hybridoma cell line stably secreting the REV gp90 MAb via cell fusion and flow cytometry. To the best of our knowledge, this study is the first to identify the novel linear B-cell epitope, ^195^REESVRERL^203^, in the REV gp90 protein.

## 5. Conclusions

Our results demonstrate that a novel, highly conserved B-cell linear epitope ^195^REESVRERL^203^ of REV gp90 protein was identified on the basis of prepared specific monoclonal antibodies, which provides critical theoretical and technical support for further exploration of REV antigen structure and establishment of novel detection methods.

## Figures and Tables

**Table 1:** The REV strains and their GenBank accession numbers used in this study.

No.	Strains	GenBank Accession No.
1	HLJR0901	GQ415646
2	HA9901	AY842951
3	ZD0708	FJ496333
4	HLJ07I	GQ375848
5	HLJR0801	GU012640
6	HLJR0902	GU012638
7	HLJR0903	GQ415643
8	HLJR0904	GU012639
9	HLJR0905	GU012643
10	JLR0801	GQ415644
11	JLR0802	GU969140
12	JLR0803	GU012644
13	JLR0901	GU012646
14	JLR0902	GQ415645
15	3122/03	DQ513316
16	3295/04	DQ513317
17	3337/05	FJ439120
18	3410/06	FJ439119
19	APC-566	DQ387450
20	170A	GU222420
21	PC-R92	DO237901
22	FA	AF246698
23	SNV	DO003591

## Data Availability

The original data presented in the study are openly available in [Science Data Bank] 800 https://www.scidb.cn/detail?dataSetId=c9be8065491143ce8afd7a3bd7653f37 (accessed on 13 January 2026)
